# Liver Biopsy for Histological Assessment: The Case in Favor

**DOI:** 10.4103/1319-3767.61245

**Published:** 2010-04

**Authors:** Khalid A. Alswat, Khalid Mumtaz, Wasim Jafri

**Affiliations:** Gastroenterology and Hepatology Division, Department of Medicine, Liver Disease Research Center, King Saud University Liver Research Center, College of Medicine, Saudi Arabia; 1 Department of Medicine, Section of Gastroenterology, Aga Khan University Hospital, Karachi, Pakistan

**Keywords:** FibroScan, FibroTest, histology, liver biopsy, markers, noninvasive assessment

## Abstract

Liver biopsy (LB) is the gold standard method for assessment of liver histology. It provides valuable, otherwise unobtainable information, regarding the degree of fibrosis, parenchymal integrity, degree and pattern of inflammation, bile duct status and deposition of materials and minerals in the liver. This information provides immense help in the diagnosis and prognostication of a variety of liver diseases. With careful selection of patients, and performance of the procedure appropriately, the complications become exceptionally rare in current clinical practice. Furthermore, the limitations of sampling error and inter-/ intra-observer variability may be avoided by obtaining adequate tissue specimen and having it reviewed by an experienced liver pathologist. Current noninvasive tools are unqualified to replace LB in clinical practice in the face of specific limitations for each tool, compounded by a poorer performance towards the assessment of the degree of liver fibrosis, particularly for intermediate stages.

Most liver diseases are usually silent except in the extremes of presentation; i.e. the clinical presentation of acute hepatitis or liver failure, and in-between exist a large spectrum of relatively silent chronic hepatitis due to different etiologies and pathophysiological processes.

Laboratory and radiological investigations may help to figure out these categories; however in many situations performing liver biopsy (LB) is essential. The value of LB is not merely to determine the degree of fibrosis, rather it draws a detailed map for many important histological findings such as the degree of inflammation, nature of inflammatory cells, distribution of inflammation, status of bile ducts, vasculature, presence of steatosis and deposition and infiltration of liver with different materials like iron, copper, etc.

Undoubtedly, this otherwise unobtainable information regarding the structural integrity of liver parenchyma, degree and type of injury and the host response, has a clear impact on the diagnosis, prognosis and response to treatment. Thus, LB has, for decades, been considered as the gold standard method for assessing liver histology.

Since the first LB performed by Paul Ehrlich in Germany in 1883,[1] this technique has proved to be a revolution in the field of hepatology. As a time-honored procedure, it has rendered landmark developments and a comprehensive understanding of various aspects of liver pathology.

LB is performed via a percutaneous approach in most clinical situations; however transjugular or laparoscopic approaches are still used in specific situations. It is usually done in an outpatient setting, requiring a few hours of post-procedure observation, and on most occasions, the patient can go back to the work on the second day.[2] Over decades, the records of LB provide evidence of simplicity and safety; however, as in the case of many clinical procedures, it is not without rare complications and limitations.

For these reasons, there has been a recent interest in developing alternative methods to study the liver histology. These methods fall broadly into two categories, either imaging techniques or serum markers. However, despite the huge resources invested in the active development and refinement of these techniques, these tests are only able to offer discrimination for the extremes of fibrosis range, with a negligible ability to provide information on other details of the pathological process.

The argument of the supporters of the use of noninvasive fibrosis markers to replace LB is summarized mainly in a few points-concerns regarding the safety of LB and the possible limitations such as sampling and interpreter variation. On the other hand, the evolving data on noninvasive markers has been accepted with an overly optimistic approach, vastly lacking the rigorous criticism reserved for new techniques and methods. We will address herein these issues in order to ascertain the validity of these arguments in the context of evolving evidence.

## ROLE OF LIVER BIOPSY

Over many decades, LB played a crucial role as a diagnostic tool for various liver diseases. Subsequently, with a better understanding of the natural history of many liver diseases and the availability of more treatment options, this role has expanded whereby LB gives valuable information for utilization in treatment decisions, and prognostication of a wide variety of liver diseases.

### Liver biopsy in diagnosis

When LB is used for diagnostic purposes, it is usually considered in conjunction with other clinical and laboratory data. Many liver diseases are diagnosed based on biochemical, serologic and sometimes genetic testing. However, some patients with conflicting or overlapping test results may still need LB for definitive diagnosis. Non-alcoholic fatty liver disease, autoimmune and cholestatic disorders, infiltrative or storage diseases, drug-induced liver injury, and some infectious, vascular and granulomatous diseases may have characteristic histological features that are helpful in diagnosis.[3] Diagnosis of overlap syndrome of primary biliary cirrhosis (PBC) and autoimmune hepatitis (AIH) requires examination of liver histology.[4] Furthermore, in daily clinical practice, the need for LB is clearer in situations where the possibility of co-existing disorders such as steatosis with hepatitis C (HCV) and hepatitis B virus (HBV) are present. The likelihood of such co-existent disorders cannot be underestimated with the current epidemic of obesity in many parts of the world.[5–7]

In a post liver transplant setting, an abnormal liver test is a frequent clinical scenario, and identifying the underlying cause is essential to the decision-making process where treatment options are being considered. Allograft rejection, drug-induced injury, bile duct or vascular injury and the recurrence of the original disease are some examples of such scenarios. Although the timing and pattern of liver test abnormalities in addition to modification of immunosuppressive regimen may help in managing some patients in this context, LB is frequently needed to resolve the ambiguity and guide further management.[8–11]

### Unexplained abnormal liver enzymes

LB is a valuable diagnostic tool in patients with chronic (>6 months) unexplained abnormal liver tests in the absence of diagnostic serology. In a study of 354 patients with abnormal liver tests and absence of diagnostic serology, 6% had a normal LB while 26% were found to have some degree of fibrosis, and another 6% were cirrhotic. Thirty four and 32% of biopsies suggested non-alcoholic steatohepatits (NASH) or fatty liver, respectively. Other diagnoses included cryptogenic hepatitis, drug toxicity, primary and secondary biliary cirrhosis, AIH, alcohol-related liver disease, primary sclerosing cholangitis, haemochromatosis, amyloid and glycogen storage disease. The management was directly altered because of LB in 18% of patients and 3 families were entered into screening programs for inheritable liver disease.[12]

In another study of 365 patients, 411 diagnoses were carried out before biopsy, 84.4% were confirmed by biopsy but in 8.8%, 6.8% and 10.5% the diagnosis was specified, changed, or a diagnosis added, respectively. In this study the authors found that LB led to change in management for 12.1% of patients.[13]

Finally, LB can help in understanding the etiology of cryptogenic cirrhosis. This category, i.e. cirrhosis of unknown etiology, is found in 3-30% of patients with cirrhosis.[14,15] NASH is considered the commonest cause of cryptogenic cirrhosis.[16–18] Other possible causes are silent or “burnt out” AIH, occult viral infection and covert alcoholism. The so-called residual histological findings such as foci of autoimmune-like inflammatory infiltrates versus NASH-like foci of steatosis, cellular ballooning, and glycogenated nuclei may help in defining the underlying cause of cryptogenic cirrhosis.[19,20]

### Liver biopsy to assess severity of liver disease and when to initiate therapy

Hepatitis B: LB is not mandatory in typical HBV cases meeting treatment criteria; however, because of the absence of curative therapy and the possible commitment of the patient to long-term therapy, it is generally advisable to assess liver histology before starting treatment to support treatment criteria by determining the degree of inflammation and fibrosis, as well as to rule out the co-existence of other conditions contributing to high transaminases. More importantly, LB has a strong influence in treatment decisions relating to HBV cases with persistently borderline, normal or slightly elevated alanine aminotransferase (ALT) levels, particularly if the patient is above the age of 40 with raised or fluctuating HBVDNA.[21,22]

Hepatitis C: Treatment can be initiated for HCV patients without LB, however, in addition to the important information obtained by LB regarding the degree of inflammation and fibrosis, examination of liver histology gives valuable information about two common non-HCV conditions which in-turn may affect disease progression and response to treatment, namely steatosis and excess hepatocellular iron deposition.[2,23]

The American Association for the Study of Liver Disease (AASLD) guidelines state that “a liver biopsy should be considered in patients with chronic hepatitis C infection if the patient and health care provider wish information regarding fibrosis stage for prognostic purposes or to make a decision regarding treatment".[24] Since the evidence is still evolving for non-invasive tests, these guidelines suggest that currently available noninvasive tests should not replace the LB in routine clinical practice.

### Safety of liver biopsy

Data on LB complications is heterogeneous with wide variation on reported rate of complications. However, experience gathered over decades from many centers, has shown a very low rate of complications [Table 1].[25–34]

**Table 1:** Summary of liver biopsy complications in various published studies

Study	Number of biopsies	Complications	Death
Pettault *et al* [25]	1,000	5.9% moderate to severe pain or hypotension or both developed	No death
Piccinino *et al* [26]	68,276	Total number of complications 147 (2.2%)	Death was 9/100,000 (only in patients with malignant diseases or cirrhosis)
Gilmore et al* [27]	1,500	1.7% bleeding (commoner when clotting was impaired or serum bilirubin raised)	0.13-0.33%
Cadranel et al** [28]	2,084	Major 0.57%	No death
Montalto *et al* [29]	1,644	One hemoperitoneum, 1 hemobilia, and 2 cases of subcapsular haematoma	One death (in hospitalized patients)
Firpi *et al* [30]	3,214	Mild to moderate pain (13%). Major complication rate was ≤1.7%	2 patients (0.06%)
Rivera-Sanfeliz *et al* [31]	154	No major complications Pain requiring analgesia-18.2%	No death
Myers et al# [32]	4,275	Pain requiring admission (0.51%) bleeding (0.35%) (most common)	Six patients (0.14%) died; all had malignancies
Howard R *et al* [33]	447	No major; minor complications-pain 32.2%, hypotension 1.3%, nausea/vomiting 0.9%	No death
Pedia *et al* [34]	539	2% (5 with severe post procedural pain, 3 with symptomatic hemorrhage, 2 with infection)	No death

* Audit by the British Society of Gastroenterology and the Royal College of Physicians of London,

** Nationwide French survey,

# Canadian population-based study

Pain is the commonest complication and rarely requires analgesia or readmission.[35,36] Bleeding is the most serious complication and rarely requires intervention or causes death. Most of the associated mortality cases are reported in patients with malignancy or advanced cirrhosis.[26,32]

Complications are related to the experience of the operator and selection of patients. However, there appears to be no effect on the rate of complications by the type of needle used.[25,37] Some evidence shows that there is no difference in the rate of complications between biopsies performed in community practice and academic institutes.[32] In addition, it is unclear whether the routine use of ultrasound, suggested by some investigators to reduce the rate of complications,[38] is cost-effective, since the value of the added benefit must be weighed against the added cost of ultrasonographic guidance.[39]

Therefore, the current evidence indicates that LB is a safe outpatient procedure, provided that patients are selected carefully and the procedure performed properly, and patient monitoring is adequate after the procedure.

### Potential sampling errors and observer variations

Since LB involves a small part of the whole liver organ, there is a risk that this might not be representative of the whole liver.[40] This risk is partly theoretical, since the inflammation and fibrosis that occurs during the course of the disease is usually diffuse and homogeneous in most of the liver diseases. Furthermore, extensive literature accumulation has shown that increasing the length of LB decreases the risk of sampling error.[41–43]

Another potential limitation of LB is the observer variation which is related to the discordance between pathologists in biopsy interpretation.[44] In addition to the small biopsy size, several factors can contributes to variation in interpretation of LB, although the level of experience (specialization, duration, and location of practice) appears to have more influence on agreement than the characteristics of the specimen (length, fibrosis class number). Hence, training and specialization of pathologists is of major importance for reducing observer variation.[45] Furthermore, the current use of histological scoring systems for evaluation of fibrosis and necro-inflammation has limited this drawback.[46,47] Thus, although LB has its limitations, appropriate precautions may reduce the flaws inherent in this method.

## LIMITATIONS OF AVAILABLE NONINVASIVE TESTS

Great efforts and strides are being made in the development of accurate noninvasive methods for the determination of fibrosis.[48] However, no single noninvasive test developed to date can provide information to match that obtained from actual histology (such as, inflammation, fibrosis, steatosis, etc).

Efforts to increase the yield of noninvasive models by combining two models of noninvasive markers have led to some increase in the accuracy of estimation of fibrosis between minimal and significant fibrosis, however, accuracy of estimation of the intermediate stages of fibrosis is still weak.[49]

In order to see if these methods are ready to replace LB, we will discuss herein two examples of noninvasive methods which have been extensively studied in different populations of liver disease and have a generally better reputation and popularity than others i.e. FibroTest and FibroScan.

### FibroTest

FibroTest (FT) is a mathematical score derived from a of group of serum markers including alpha-2-macroglobulin, gamma-glutamyl-transpeptidase, haptoglobin, apolipoprotein-A1, and total bilirubin, in addition to patient age and gender using a patented algorithm.[50] This score has been shown to have a higher diagnostic area under the receiver operating characteristic (AUROC) curve than other biochemical markers, including hyaluronic acid (HA), the Forn's index, and the AST/platelet ratio index (APRI).[51] A study by a French group showed AUROC for significant fibrosis (F2-F4), and severe fibrosis (F3-F4) were 0.79 [0.75-0.82], and 0.80 [0.76-0.83], respectively.[52] The same conclusion was not reached upon by an Australian group. In their study, Rossi *et al* found 33 of the 125 patients had FT scores <0.1 and were therefore deemed unlikely to have fibrosis, but 6 (18%) of these had significant fibrosis on histology. Conversely, of the 24 patients with scores >0.6 who were likely to have significant fibrosis, 5 (21%) had mild fibrosis on histology.[53]

FT has been assessed mainly in HCV, with recent reports arising in other liver diseases including HBV, NAFLD and ALD. In a metaanlysis of 38 diagnostic studies which pooled 7985 subjects who had undergone both FT and biopsy (4600 HCV, 1580 HBV, 267 NAFLD, 524 ALD and 1014 mixed). The mean standardized AUROC for the diagnosis of bridging fibrosis (F2/F3/F4 vs. F0/F1) was 0.84 (95% CI, 0.83-0.86), with no differences in terms of causes of liver disease: HCV 0.84 (0.82-0.87); HBV 0.81 (0.78-0.83); NAFLD 0.84 (0.76-0.92); ALD 0.87 (0.82-0.92); and mixed 0.85 (0.81-0.89).[54]

The inter-laboratory variations which are in the quality controlled, analytically acceptable range may have impact on the result of FT and can lead to significant discordance between histology and FT score.[55] In addition, FT has some other limitations in several medical conditions, such as Gilbert's syndrome, hemolysis, renal failure, inflammatory conditions and biliary obstruction.[56] More independent validation of FT in Hepatitis C and other less studied diseases is required since there are only a few studies that have assessed FT other than the French study group pioneering research in this area. FT has no up-front cost, but is associated with recurring cost for each use, whereupon the test may prove costly if utilized for follow up and monitoring. Finally, there is a significant delay entailed in reporting of the results of the biochemical tests that form part of the mathematical model of FT.

### Concerns regarding serum markers

It is worthwhile to consider some of the concerns pertaining to FT and other serum markers regarding adoption in routine clinical practice in the assessment of liver fibrosis instead of LB.[57,58] Firstly, none of these serum markers is liver specific, and reflect the inflammatory process and not fibrosis.

Secondly, these markers have a relatively good prediction for the extremes of fibrosis stage, which may help sometimes in the treatment decision, but nonetheless, have a poor performance in correctly classifying the intermediate stages. Therefore, this factor will limit their use in longitudinal studies for the purpose of studying liver fibrosis progression, especially if their results were accepted for treatment decision.

Additionally, these markers need to be validated in different clinical settings. The rationale is that the predictive value of any test is affected by the prevalence of the disease. So the clinical utility of these markers is critically affected by the prevalence of fibrosis in the population being investigated and almost all published studies have been performed in a tertiary-care setting.

Moreover, it is likely that the result of these markers, whether used individually or in combination, will be affected by the difference in the assays used. At present, the recommendation is that the methods used to measure serum markers should be identical to those reported in the original publication. However, as these tests become routinely available and regularly performed in real-life practice, it is possible that these aspects will be brought into greater focus.

Finally, a major shortcoming of the existing literature is the failure to demonstrate the cost-effectiveness of these measures. This is vital since erroneous results may lead to unnecessary treatment of patients with mild disease or withholding of treatment from those with advanced disease who may go on to develop costly complications. Moreover, costs are likely to be inflated due to the tendency of physicians to perform repeated assessments using these noninvasive measures (e.g., up to every 6-12 months as recommended by the FT developers).

### FibroScan

FibroScan (FS) or transient elastography is a novel technique for measurement of liver stiffness. This is a rapid, non- invasive technique that utilises low frequency vibration and ultrasound to assess the stiffness of liver tissue. FS has good prediction of cirrhosis however, performance is less in mild to intermediate stages of fibrosis.[59]

A recent systematic review evaluated the accuracy of FT and FS in HCV patients. The AUROCs for the prediction of significant fibrosis (stages 2-4) for FT and FS were 0.81 (95% [confidence interval] CI: 0.78-84) and 0.83 (95% CI: 0.03-1.00), respectively. The sensitivity and specificity of FT at a threshold of 0.60, were 47% (35-59%) and 90% (87-92%). For FS (threshold approximately 8 kPa), corresponding values were 64% (50-76%) and 87% (80-91%), respectively. Better result was obtained for prediction of cirrhosis, with the AUROCs for FS and FS were 0.90 (95% CI not calculable) and 0.95 (0.87-0.99), respectively.[60] Discordance of at least two stages between transient elastography and histological assessment were observed in 28 (11%) of the 254 consecutive patients with liver biopsy of at least 15 mm, in a multivariate analysis, fibrosis stage (F0-F2 versus F3-F4) and the ratio interquartile range/median value of liver stiffness measurement (IQR/M) were associated with discordances (*P*<0.05).[61]

The cause-specific cut-off values need to be ascertained, since the best predicting cut-off values vary according to the etiology. For instance, the cut-off value for the diagnosis of HCV cirrhosis is 12.5 kPa, 19 to 21.5 kPa in alcoholic cirrhosis, 17.5 kPa in NASH cirrhosis and 17.3 kPa for cirrhosis secondary to primary sclerosing cholangitis or primary biliary cirrhosis.[62]

FS has some limitations in addition to its poorer performance in patients with mild to moderate fibrosis. This technique cannot be used in patients with ascites because the poor propagation of elastic shear waves through liquid. It also performs poorly in morbidly obese patients, since adipose tissue attenuates both shear waves and ultrasound waves. Other conditions such as steatosis, cholestasis and liver congestion due to heart failure may affect the accuracy of the result.[59,63]

The failure rates range between 2.4% and 9.4%.[64,65] this being mainly in obese patients and in those with narrow intercostal spaces. In multivariate analysis, the only factor associated with failure of FS was a body mass index >28 kg/m^2^ (odds ratio 10.0; 95% CI: 5.7-17.9, *P*=0.001).[66]

FS frequently yields pathologically high values in patients with acute liver damage, or at the time of an ALT flare, and is unsuitable for detecting cirrhosis/fibrosis in this scenario. In 15 of 20 patients with acute liver damage mostly due to HBV and drugs, with serum ALT activities ranging from 151 to 5382 U/L (mean: 1355±1217 U/L), initial liver stiffness values measured by FS during the acute phase of the liver damage were suggestive of liver cirrhosis. However, none of these 15 patients showed any signs of liver cirrhosis in the physical examination, ultrasound examination, or liver histology (performed in 11 of 15 [73%] patients). Six patients with initially high liver stiffness were followed up to abatement of the acute hepatitic phase; in all of them, liver stiffness values decreased to values below the cut-off level for liver cirrhosis.[67]

Furthermore, the position of the probe may impact the accuracy and reproducibility of this technique. Among 268 patients with both anterior and reference positions, the FS measurements estimated at the reference position (9.0 kPa; [0.5]) was significantly higher in comparison to the anterior position (8.5 kPa [0.5]; *P*<0.0001).[68]

Reproducibility of FS is generally good, however the interobserver agreement is significantly reduced in some situations such as lower degrees of hepatic fibrosis (intraclass correlation coefficients (ICC) for F0-F1 0.60 versus 0.99 for F≥2), hepatic steatosis (ICC for steatosis ≥25% of hepatocytes 0.90 versus 0.98 for <25%) and increased BMI (ICC for BMI≥25 kg/m^2^ 0.94 versus 0.98 for <25 kg/m^2^).[64] Although, there is no recurring cost of the FS machine, the upfront cost is relatively high, which will limit its widespread use through many secondary and tertiary care centers.

In conclusion, considering the value and safety of LB, and the current limitations of noninvasive tests, LB will continue to remain, in the foreseeable future, as the cornerstone and the gold standard test in the assessment of liver fibrosis and histology. While great efforts and advances have been made to improve noninvasive markers, nevertheless, major steps remain ahead before these tests can replace LB in both the information obtained and the accuracy in diagnosis.

## References

[1] Schiff ER, Sorrell MF, Willis C (2003). Schiff's Diseases of the Liver textbook.

[2] Rockey DC, Caldwell SH, Goodman ZD, Nelson RC, Smith AD (2009). Liver biopsy. Hepatology.

[3] Czaja AJ, Carpenter HA (2007). Optimizing diagnosis from the medical liver biopsy. Clin Gastroenterol Hepatol.

[4] Chazouillères O, Wendum D, Serfaty L, Montembault S, Rosmorduc O, Poupon R (1998). Primary biliary cirrhosis-autoimmune hepatitis overlap syndrome: Clinical features and response to therapy. Hepatology.

[5] Powell EE, Jonsson JR, Clouston AD (2005). Steatosis: Co-factor in other liver diseases. Hepatology.

[6] Schwimmer JB (2007). Definitive diagnosis and assessment of risk for nonalcoholic fatty liver disease in children and adolescents. Semin Liver Dis.

[7] Caballería L, Pera G, Auladell MA, Torán P, Muñoz L, Miranda D (2010). Prevalence and factors associated with the presence of nonalcoholic fatty liver disease in an adult population in Spain. Eur J Gastroenterol Hepatol.

[8] Petrovic LM, Villamil FG, Vierling JM, Makowka L, Geller SA (1997). Comparison of histopathology in acute allograft rejection and recurrent hepatitis C infection after liver transplantation. Liver Transpl Surg.

[9] Ferrell LD, Wright TL, Roberts J, Ascher N, Lake J (1992). Hepatitis C viral infection in liver transplant recipients. Hepatology.

[10] Aran PP, Bissel MG, Whitington PF, Bostwick DG, Adamac T, Baker AL (1993). Diagnosis of hepatic allograft rejection: Role of liver biopsy. Clin Transplant.

[11] Yu YY, Ji J, Zhou GW, Shen BY, Chen H, Yan JQ (2004). Liver biopsy in evaluation of complications following liver transplantation. World J Gastroenterol.

[12] Skelly MM, James PD, Ryder SD (2001). Findings on liver biopsy to investigate abnormal liver function tests in the absence of diagnostic serology. J Hepatol.

[13] Spycher C, Zimmermann A, Reichen J (2001). The diagnostic value of liver biopsy. BMC Gastroenterol.

[14] Byron D, Minuk GY (1996). Clinical hepatology: Profile of an urban, hospital based practice. Hepatology.

[15] Greeve M, Ferrell L, Kim M, Combs C, Roberts J, Ascher N (1993). Cirrhosis of undefined pathogenesis: Absence of evidence for unknown viruses or autoimmune processes. Hepatology.

[16] Caldwell SH, Oelsner DH, Iezzoni JC, Hespenheide EE, Battle EH, Driscoll CJ (1999). Cryptogenic cirrhosis: Clinical characterization and risk factors for underlying disease. Hepatology.

[17] Poonawala A, Nair SP, Thuluvath PJ (2000). Prevalence of obesity and diabetes in patients with cryptogenic cirrhosis: A case-control study. Hepatology.

[18] Kojima H, Sakurai S, Matsumura M, Umemoto N, Uemura M, Morimoto H (2006). Cryptogenic cirrhosis in the region where obesity is not prevalent. World J Gastroenterol.

[19] Contos MJ, Cales W, Sterling RK, Luketic VA, Shiffman ML, Mills AS (2001). Development of nonalcoholic fatty liver disease after orthotopic liver transplantation for cryptogenic cirrhosis. Liver Transpl.

[20] Ayata G, Gordon FD, Lewis WD, Pomfret E, Pomposelli JJ, Jenkins RL (2002). Cryptogenic cirrhosis: Clinicopathologic findings at and after liver transplantation. Hum Pathol.

[21] Mani H, Kleiner DE (2009). Liver biopsy findings in chronic hepatitis B. Hepatology.

[22] Keeffe EB, Dieterich DT, Han SH, Jacobson IM, Martin P, Schiff ER (2008). A treatment algorithm for the management of chronic hepatitis B virus infection in the United States: 2008 update. Clin Gastroenterol Hepatol.

[23] Gebo KA, Herlong HF, Torbenson MS, Jenckes MW, Chander G, Ghanem KG (2002). Role of liver biopsy in management of chronic hepatitis C: A systematic review. Hepatology.

[24] Ghany MG, Strader DB, Thomas DL, Seeff LB (2009). Diagnosis, management, and treatment of hepatitis C: An update. Hepatology.

[25] Perrault J, McGill DB, Ott BJ, Taylor WF (1978). Liver biopsy: Complications in 1000 inpatients and outpatients. Gastroenterology.

[26] Piccinino F, Sagnelli E, Pasquale G, Giusti G (1986). Complications following percutaneous liver biopsy: A multicentre retrospective study on 68,276 biopsies. J Hepatol.

[27] Gilmore IT, Burroughs A, Murray-Lyon IM, Williams R, Jenkins D, Hopkins A (1995). Indications, methods, and outcomes of percutaneous liver biopsy in England and Wales: An audit by the British Society of Gastroenterology and the Royal College of Physicians of London. Gut.

[28] Cadranel JF, Rufat P, Degos F (2000). Practices of liver biopsy in France: Results of a prospective nationwide survey: For the Group of Epidemiology of the French Association for the Study of the Liver (AFEF). Hepatology.

[29] Montalto G, Soresi M, Carroccio A, Bascone F, Tripi S, Aragona F (2001). Percutaneous liver biopsy: a safe outpatient procedure?. Digestion.

[30] Firipi RJ, Soldevila-Pico C, Abdelmalek MF, Morelli G, Judah J, Nelson DR (2005). Short recovery time after percutaneous liver biopsy: Should we change our current practices?. Clin Gastroenterol Hepatol.

[31] Rivera-Sanfeliz G, Kinney TB, Rose SC, Agha AK, Valji K, Miller FJ (2005). Single-pass percutaneous liver biopsy for diffuse liver disease using an automated device: experience in 154 procedures. Cardiovasc Intervent Radiol.

[32] Myers RP, Fong A, Shaheen AA (2008). Utilization rates, complications and costs of percutaneous liver biopsy: A population-based study including 4275 biopsies. Liver Int.

[33] Howard R, Karageorge G, van Harselaar K, Bell M, Basford P, Schultz M (2008). Post-procedure surveillance in liver biopsy: How long is long enough?. N Z Med J.

[34] Padia SA, Baker ME, Schaeffer CJ, Remer EM, Obuchowski NA, Winans C (2009). Safety and efficacy of sonographic-guided random real-time core needle biopsy of the liver. J Clin Ultrasound.

[35] Janes CH, Lindor KD (1993). Outcome of patients hospitalized for complications after outpatient liver biopsy. Ann Intern Med.

[36] Weigand K, Weigand K (2009). Percutaneous liver biopsy: Retrospective study over 15 years comparing 287 inpatients with 428 outpatients. J Gastroenterol Hepatol.

[37] Froehlich F, Lamy O, Fried M, Gonvers JJ (1993). Practice and complications of liver biopsy results of a nationwide survey in Switzerland. Dig Dis Sci.

[38] Farrell RJ, Smiddy PF, Pilkington RM, Tobin AA, Mooney EE, Temperley IJ (1999). Guided versus blind liver biopsy for chronic hepatitis C: Clinical benefits and costs. J Hepatol.

[39] Lindor KD, Bru C, Jorgensen RA, Rakela J, Bordas JM, Gross JB (1996). The role of ultrasonography and automatic-needle biopsy in outpatient percutaneous liver biopsy. Hepatology.

[40] Regev A, Berho M, Jeffers LJ, Milikowski C, Molina EG, Pyrsopoulos NT (2002). Sampling error and intraobser ver variation in liver biopsy in patients with chronic HCV infection. Am J Gastroenterol.

[41] Schiano TD, Azeem S, Bodian CA, Bodenheimer HC, Merati S, Thung SN (2005). Importance of specimen size in accurate needle liver biopsy evaluation of patients with chronic hepatitis C. Clin Gastroenterol Hepatol.

[42] Bedossa P, Dargere D, Paradis V (2003). Sampling variability of liver fibrosis in chronic hepatitis C. Hepatology.

[43] Colloredo G, Guido M, Sonzogni Aleandro G (2003). Impact of liver biopsy size on histological evaluation of chronic viral hepatitis: The smaller the sample, the milder the disease. J Hepatol.

[44] Skripenova S, Trainer TD, Krawitt EL, Blaszyk H (2007). Variability of grade and stage in simultaneous paired liver biopsies in patients with hepatitis C. J Clin Pathol.

[45] Rousselet MC, Michalak S, Dupré F, Croué A, Bedossa P, Saint-André JP (2005). Sources of variability in histological scoring of chronic viral hepatitis. Hepatology.

[46] Grønbaek K, Christensen PB, Hamilton-Dutoit S, Federspiel BH, Hage E, Jensen OJ (2002). Interobserver variation in interpretation of serial liver biopsies from patients with chronic hepatitis C. J Viral Hepat.

[47] Goldin RD, Goldin JG, Burt AD, Dhillon PA, Hubscher S, Wyatt J (1996). Intra-observer and inter-observer variation in the histopathological assessment of chronic viral hepatitis. J Hepatol.

[48] Poynard T, Morra R, Ingiliz P, Imbert-Bismut F, Thabut D, Messous D (2008). Assessment of liver fibrosis: Noninvasive means. Saudi J Gastroenterol.

[49] Smith JO, Sterling RK (2009). Systematic review: Non-invasive methods of fibrosis analysis in chronic hepatitis C. Aliment Pharmacol Ther.

[50] Imbert-Bismut F, Ratziu V, Pieroni L, Charlotte F, Benhamou Y, Poynard T (2001). Biochemical markers of liver fibrosis in patients with hepatitis C virus infection: A prospective study. Lancet.

[51] Poynard T, Imbert-Bismut F, Munteanu M, Messous D, Myers RP, Thabut D (2004). Overview of the diagnostic value of biochemical markers of liver fibrosis (FibroTest, HCV FibroSure) and necrosis (ActiTest) in patients with chronic hepatitis C. Comp Hepatol.

[52] Halfon P, Bourliere M, Deydier R, Botta-Fridlund D, Renou C, Tran A (2006). Independent prospective multicenter validation of biochemical markers (fibrotest-actitest) for the prediction of liver fibrosis and activity in patients with chronic hepatitis C: The fibropaca study. Am J Gastroenterol.

[53] Rossi E, Adams L, Prins A, Bulsara M, de Boer B, Garas G (2003). Validation of the FibroTest biochemical markers score in assessing liver fibrosis in hepatitis C patients. Clin Chem.

[54] Halfon P, Munteanu M, Poynard T (2008). FibroTest-ActiTest as a non-invasive marker of liver fibrosis. Gastroenterol Clin Biol.

[55] Gressner OA, Beer N, Jodlowski A, Gressner AM (2010). Impact of quality control accepted inter-laboratory variations on calculated Fibrotest/ Actitest scores for the non-invasive biochemical assessment of liver fibrosis. Clin Chim Acta.

[56] Poynard T, Munteanu M, Imbert-Bismut F, Charlotte F, Thabut D, Le Calvez S (2004). Prospective analysis of discordant results between biochemical markers and biopsy in patients with chronic hepatitis C. Clin Chem.

[57] Bedossa P (2009). Assessment of hepatitis C: Non-invasive fibrosis markers and/or liver biopsy. Liver Int.

[58] Oh S, Afdhal NH (2001). Hepatic fibrosis: Are any of the serum markers useful?. Curr Gastroenterol Rep.

[59] Ziol M, Handra-Luca A, Kettaneh A, Christidis C, Mal F, Kazemi F (2005). Noninvasive assessment of liver fibrosis by measurement of stiffness in patients with chronic hepatitis C. Hepatology.

[60] Shaheen AA, Wan AF, Myers RP (2007). FibroTest and FibroScan for the prediction of hepatitis C-related fibrosis: A systematic review of diagnostic test accuracy. Am J Gastroenterol.

[61] Lucidarme D, Foucher J, Le Bail B, Vergniol J, Castera L, Duburque C (2009). Factors of accuracy of transient elastography (fibroscan) for the diagnosis of liver fibrosis in chronic hepatitis C. Hepatology.

[62] Nguyen-Khac E, Capron D, Braillon A, Dupas JL (2008). The non-invasive diagnosis of cirrhosis using the Fibroscan must be performed with cause-specific stiffness cut-offs. Gut.

[63] Vizzutti F, Arena U, Marra F, Pinzani M (2009). Elastography for the non-invasive assessment of liver disease: Limitations and future developments. Gut.

[64] Fraquelli M, Rigamonti C, Casazza G, Conte D, Donato MF, Ronchi G (2007). Reproducibility of transient elastography in the evaluation of liver fibrosis in patients with chronic liver disease. Gut.

[65] Ganne-Carrié N, Ziol M, de Ledinghen V, Douvin C, Marcellin P, Castera L (2006). Accuracy of liver stiffness measurement for the diagnosis of cirrhosis in patients with chronic liver diseases. Hepatology.

[66] Foucher J, Castéra L, Bernard PH, Adhoute X, Laharie D, Bertet J (2006). Prevalence and factors associated with failure of liver stiffness measurement using FibroScan in a prospective study of 2114 examinations. Eur J Gastroenterol Hepatol.

[67] Sagir A, Erhardt A, Schmitt M, Häussinger D (2008). Transient elastography is unreliable for detection of cirrhosis in patients with acute liver damage. Hepatology.

[68] Ingiliz P, Chhay KP, Munteanu M, Lebray P, Ngo Y, Roulot D (2009). Applicability and variability of liver stiffness measurements according to probe position. World J Gastroenterol.

